# The importance of data quality for generating reliable distribution models for rare, elusive, and cryptic species

**DOI:** 10.1371/journal.pone.0179152

**Published:** 2017-06-22

**Authors:** Keith B. Aubry, Catherine M. Raley, Kevin S. McKelvey

**Affiliations:** 1 United States Department of Agriculture, Forest Service, Pacific Northwest Research Station, Olympia, Washington, United States of America; 2 United States Department of Agriculture, Forest Service, Rocky Mountain Research Station, Missoula, Montana, United States of America; Clemson University, UNITED STATES

## Abstract

The availability of spatially referenced environmental data and species occurrence records in online databases enable practitioners to easily generate species distribution models (SDMs) for a broad array of taxa. Such databases often include occurrence records of unknown reliability, yet little information is available on the influence of data quality on SDMs generated for rare, elusive, and cryptic species that are prone to misidentification in the field. We investigated this question for the fisher (*Pekania pennanti*), a forest carnivore of conservation concern in the Pacific States that is often confused with the more common Pacific marten (*Martes caurina*). Fisher occurrence records supported by physical evidence (verifiable records) were available from a limited area, whereas occurrence records of unknown quality (unscreened records) were available from throughout the fisher’s historical range. We reserved 20% of the verifiable records to use as a test sample for both models and generated SDMs with each dataset using Maxent. The verifiable model performed substantially better than the unscreened model based on multiple metrics including AUC_test_ values (0.78 and 0.62, respectively), evaluation of training and test gains, and statistical tests of how well each model predicted test localities. In addition, the verifiable model was consistent with our knowledge of the fisher’s habitat relations and potential distribution, whereas the unscreened model indicated a much broader area of high-quality habitat (indices > 0.5) that included large expanses of high-elevation habitat that fishers do not occupy. Because Pacific martens remain relatively common in upper elevation habitats in the Cascade Range and Sierra Nevada, the SDM based on unscreened records likely reflects primarily a conflation of marten and fisher habitat. Consequently, accurate identifications are far more important than the spatial extent of occurrence records for generating reliable SDMs for the fisher in this region. We strongly recommend that practitioners avoid using anecdotal occurrence records to build SDMs but, if such data are used, the validity of resulting models should be tested with verifiable occurrence records.

## Introduction

The increased availability of online satellite imagery and biodiversity databases have greatly expanded the use of spatial data in science and conservation. In particular, the broad availability of spatially contiguous environmental data and spatially referenced species occurrence records have enabled researchers to generate species distribution models (SDMs) from datasets that are readily available on the internet [1–3]. Recently, maximum entropy (Maxent) modeling has become one of the most common methods for generating SDMs, due to its use of presence-only data, its strong performance compared to other approaches [3–5], and the availability of user-friendly computer software for generating Maxent models [6].

Species occurrence records suffer from 3 primary sources of error that may affect the performance and reliability of resulting Maxent models: inaccurate spatial locations, biased sampling, and misidentifications [7]. Maxent models are generally robust to moderate spatial errors (i.e., < 5 km) in occurrence records [5, 8–9] and to small sample sizes [3, 10], but are sensitive to non-representative sampling within the analysis area, and the clustering of records in areas that are easily accessed or where target animals are more detectable, such as along roads or trails [11–14]. In contrast, the influence of misidentifications on the performance and reliability of resulting Maxent models has received much less attention.

Environmental covariate layers used to generate Maxent models are generally derived from reliable sources, but public species-occurrence databases (e.g., Global Biodiversity Information Facility [GBIF], NatureServe, biodiversity databases maintained by state and federal resource agencies) are compiled opportunistically, and typically include both highly reliable occurrence records that are associated with physical evidence, and anecdotal observations of widely varying reliability made by individuals with unknown qualifications [15]. For the generation of SDMs, it is important to recognize that occurrence records contained in such databases have not been obtained using consistent methodologies in a spatially representative manner and, more importantly, are likely to contain misidentifications [16]. Of particular concern for those interested in generating Maxent models for rare and elusive organisms, misidentifications can dominate such databases [17].

To demonstrate the ease with which Maxent can be used to generate ecologically compelling SDMs with high predictive power from unreliable occurrence data, Lozier et al. [18] compared Maxent models generated for the cryptozoid Sasquatch using occurrence records contained in a repository of putative sightings, auditory detections, and tracks (i.e., anecdotal records lacking physical evidence), and for the American black bear (*Ursus americanus*) using only specimen records contained in the GBIF (i.e., highly reliable records associated with physical evidence). Maxent models for both Sasquatch and black bear performed extremely well (the area under the receiver operating characteristic curve for test data [AUC_test_] was > 0.98), predicted distributions were strikingly similar in geographic extent, aligned well with general knowledge of each “species” range and habitat associations, and the same bioclimatic variables contributed the most to both models. The authors concluded that black bears were likely the primary “contaminating” species in the Sasquatch model (i.e., that most Sasquatch records were misidentifications of black bears), and recommended that the reliability of occurrence records be scrutinized carefully before including them in SDMs.

Using occurrence records from several online databases, the primary literature, and field observations for a taxonomically challenging invasive plant species, the yellowdevil hawkweed (*Pilosella glomerata*), Ensing et al. [19] evaluated how Maxent models varied in relation to differences in the reliability of occurrence records. Two models were generated and compared; one using a taxonomically reliable and geographically restricted dataset, and another using all available records, which included the subset of reliable records as well as many records of unknown reliability from a much broader geographic area. Both models performed extremely well (AUC_test_ > 0.94), but the predicted distribution for the “reliable records” model was 88% smaller and more homogeneous ecologically than the “all available records” model, indicating the extent to which misidentifications in occurrence records can confound the interpretation and application of SDMs for management or conservation. Similar findings were reported by Costa et al. [20], who used simulated datasets to generate a series of Maxent models that included occurrence records for both a “target” and a “contaminating” species, with misidentification rates ranging from 1 to 32%. The magnitude and direction of changes to predicted distributions were positively related to both the misidentification rate and ecological differences between the target and contaminating species.

To investigate the potential effects of misidentifications and varying spatial precision on the performance of Maxent models for the white-nosed coati (*Nasua narica*) in the American Southwest, Frey et al. [7] generated a series of models using either bioclimatic or biophysical environmental covariates. For each set of covariates, they generated 7 Maxent models using occurrence records that varied either in the reliability of species identifications or in their spatial precision. They ranked the reliability of species identifications based on the presence or absence of physical evidence, the qualifications of the observer, the details provided, and environmental conditions when the observation was made. All resulting models performed very well (AUC_test_ > 0.87), however, even those that included species identifications considered to be “highly questionable”, and coordinates that were > 3 km from the actual location. As others have reported [5, 8–9], the Maxent models they generated were robust to moderately large location errors in occurrence records. However, because the coati’s daily activities are primarily diurnal, and their appearance and behavior are highly distinctive, they are not prone to being misidentified by observers [7]. Consequently, it is likely that misidentifications were relatively uncommon in the occurrence datasets they used, even those they considered to be potentially unreliable.

It remains unclear how the quality of occurrence records affects the performance and reliability of Maxent models generated for species that are rare, elusive, and prone to misidentification. One such species is the fisher (*Pekania pennanti*), a forest-dwelling mesocarnivore of conservation concern in the Pacific States (Washington, Oregon, and California) [21–22] that was sympatric or parapatric with the Pacific marten (*Martes caurina*) in many portions of its historical range [23]. Pacific martens are relatively common in the Cascade Range and Sierra Nevada [24], whereas fishers are extirpated or have declined precipitously throughout much of this region [16]. Martens are similar to fishers in size, shape, coloration, and ecological affinities, and even experienced biologists can mistake them for fishers ([21, 25]; K. Aubry unpublished data).

Species distribution models are becoming increasingly important for fisher conservation efforts in the Pacific States [26–30]. Because fisher occurrence records associated with physical evidence are scarce [16], practitioners may be tempted to use records of unknown quality contained in public databases to generate SDMs for the fisher. Given the possibility that martens or other species were misidentified as fishers in such databases, it is important to gain a better understanding of how data quality influences the performance and reliability of resulting SDMs. To investigate this question, we compared the performance and reliability of SDMs generated for the fisher in the Pacific States with Maxent using datasets that contrasted strongly in data quality.

## Materials and methods

### Fisher occurrence records

**Fig 1:**
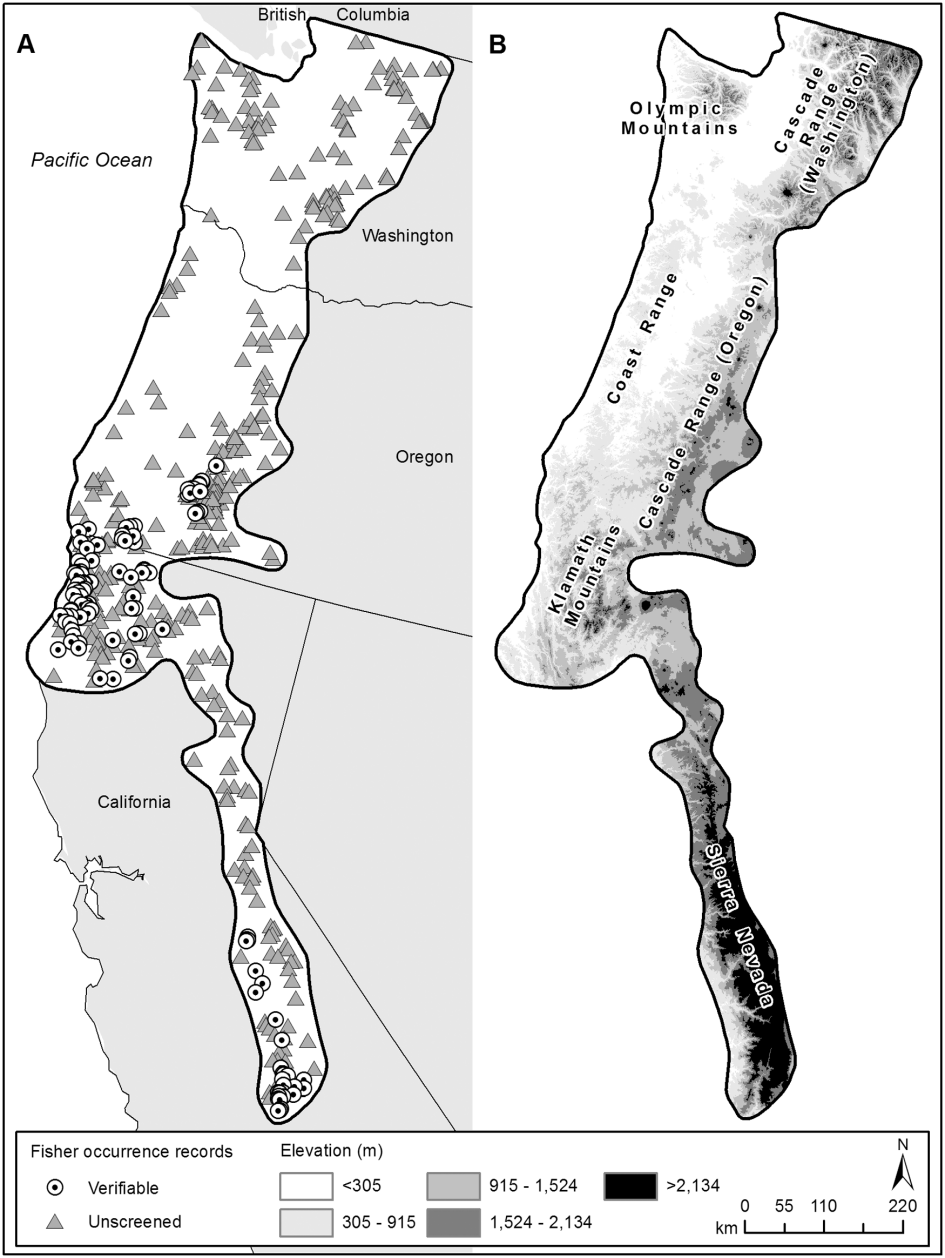
Maps depicting (A) the geographic extent of our analysis area in the Pacific States and the locations of verifiable and unscreened fisher occurrence records used in Maxent modeling, and (B) the elevational gradient and major physiographic regions that occur within our analysis area.

We used 2 independent sets of fisher occurrence records from the Pacific States compiled by Aubry and Lewis [16] to generate species distribution models in Maxent (Table 1). The first dataset included 125 fisher occurrence records obtained from 1989 to 2001 with highly reliable species identifications that were associated with physical evidence and subject to independent verification (i.e., remote-camera or track-plate detections, opportunistic photographs or digital images, incidental captures, and road-kills; hereafter, verifiable records). The second dataset included 389 fisher occurrence records obtained from 1954 to 1993 of varying and largely unknown reliability (hereafter, unscreened records). Unscreened records consisted primarily of anecdotal observations compiled by state and federal resource management agencies, and the results of questionnaires sent to registered trappers or hound hunters. To generate species distribution models, Maxent assumes a uniform probability distribution among the background samples; consequently, it is sensitive to the extent of the analysis area [6, 31]. Our analysis area encompassed approximately 262,900 km^2^, and was delineated using a 15-km buffer around all verifiable and unscreened fisher occurrence records in western Washington, western Oregon, and California (Fig 1A). The geographic extent of our analysis area corresponds closely to the historical range of fishers in the Pacific States [23].

**Table 1:** Fisher occurrence records from the Pacific States used to evaluate the effects of data quality on the performance and reliability of Maxent species distribution models.

Data Set	Region	Year	*n*	References
Verifiable records	Western Washington	1990–1997	0^a^	[32]
	Western Oregon	1994–2001	29	[16]
	California	1989–1994	96	[25]
Unscreened records	Western Washington	1955–1992	94^b^	[33–34]; K. Aubry, unpublished data
	Western Oregon	1954–1993	145^c^	[16, 34]
	California	1960–1987	150	[35–36]

^a^A series of standardized remote-camera surveys involving > 17,000 sample nights were conducted throughout western Washington by various researchers from 1990 to 1997; no fishers were detected.

^b^We excluded 22 unscreened fisher occurrence records from Washington compiled by Aubry and Houston [33] because they had spatial precision > 5.5 km or were located in different ecoregions [34] outside our analysis area, and included 1 additional record from 1992 that was not reported by Aubry and Houston [33].

^c^We excluded 11 unscreened fisher occurrence records in Oregon compiled by Aubry and Lewis [16] because they were located in different ecoregions [34] outside our analysis area.

The verifiable records we used in our analyses were unlikely to contain misidentifications but were limited in distribution (Fig 1A). In contrast, unscreened records varied substantially in the reliability of species identifications, but occurred throughout the historical range of fishers (Fig 1A). Only occurrence records that could be plotted on a map within an area < 93.2 km^2^ (1 township; see [16]) were used in our analyses. Thus, the estimated spatial precision of occurrence records in both datasets was < 5.5 km (i.e., coordinates were within 5.5 km of the actual location). Because spatial errors of this magnitude have been shown to have little effect on the performance of Maxent species distribution models [5, 7–9], differences in model performance between these datasets should be attributable primarily to species misidentifications and the geographic extent of each dataset. Accordingly, these 2 datasets enable us to evaluate the effects of both species misidentifications and geographically restricted sampling on the performance and reliability of Maxent species distribution models for a rare, elusive, and cryptic forest carnivore.

### Environmental data

We used published information on the habitat relations of fishers in western North America and our knowledge and experience to identify a suite of 8 environmental covariates that were likely to influence the distribution of fishers in the Pacific States (Table 2). In western North America, fishers are associated with low- to mid-elevation forests (generally < 1,250 m) where deep, soft snow does not accumulate, with relatively dense canopies and high structural diversity, including abundant large live trees, snags, and logs [16, 21, 37–38]. Using gradient nearest neighbor (GNN) raster data derived from 2012 Landsat imagery and inventory plots [39], we selected 5 vegetative covariates that represented varying forest types, stand ages, and structural conditions: VEGCLASS (vegetation class), AGE (stand age), CANCOV (canopy cover), DDI (diameter diversity index), and QMD (quadratic mean diameter). We also included BIOMASS (forest biomass) derived by the USDA Forest Service’s Forest Inventory and Analysis program and Remote Sensing Applications Center [40], because several studies have shown that indices of primary productivity were important contributors to fisher distribution models [26–28]. Travelling in deep, soft snow is energetically demanding for fishers [41–42], and topography has been shown to influence fisher habitat selection through its effects on microclimate, forest growth, and other processes [28, 43–44]. Accordingly, we also included SNOW (winter snowfall) and TPI (topographic position index) in our analyses. We calculated TPI within a circular neighborhood (2-km radius) using Land Facet Corridor Designer for ArcGIS 10 [45]. Detailed descriptions of how we derived each covariate are presented in Table 2. We used ArcGIS 10.2.2 to prepare all environmental raster data for Maxent modeling and, if necessary, to resample grids to the resolution of our analyses (250 x 250-m pixels; see Table 2).

**Table 2:** Environmental covariates considered for generating species distribution models for the fisher in the Pacific States using Maxent. The 6 covariates selected for Maxent modeling are shaded gray.

Category	Covariate	Reference	Description
Climatic	Winter snowfall (SNOW)	[46]	Average precipitation (mm) as snow during winter (Dec-Feb) from 1971 to 2000. Derived from 4 x 4-km PRISM data and downscaled to 250 x 250-m pixels.
Topographic	Topographic position index (TPI)	[47]	Estimate of terrain ruggedness, based on differences in elevation between a cell and the cells in the surrounding neighborhood. Negative values indicate valleys or canyon bottoms, and positive values indicate ridges or hilltops. Calculated from a 250 x 250-m digital elevation model.
Vegetative	Forest biomass (BIOMASS)	[40]	Aboveground live forest biomass (mg/ha). Used at the original resolution of 250 x 250-m pixels.
	Vegetation class (VEGCLASS)	[39]	Forest vegetation classes based on canopy cover, basal area of hardwoods, and quadratic mean diameter of dominant and codominant trees. Derived from 30 x 30-m raster data resampled to 250 x 250-m pixels using a majority algorithm.
	Stand age (AGE)	[39]	Basal area weighted stand age (year). Derived from 30 x 30-m raster data resampled to 250 x 250-m pixels using bilinear interpolation.
	Canopy cover (CANCOV)	[39]	Canopy cover of all live trees (%). Derived from 30 x 30-m raster data resampled to 250 x 250-m pixels using bilinear interpolation.
	Diameter diversity index (DDI)	[39]	Diameter diversity index of forest stands based on tree densities in different DBH classes. Derived from 30 x 30-m raster data resampled to 250 x 250-m pixels using bilinear interpolation.
	Quadratic mean diameter (QMD)	[39]	Quadratic mean diameter of all dominant and codominant trees (cm). Derived from 30 x 30-m raster data resampled to 250 x 250-m pixels using bilinear interpolation.

### Modeling

To generate fisher distribution models, we used Maximum Entropy Species Distribution Modeling software (Maxent 3.3.3k; [48]). Prior to model development, we excluded DDI and QMD from our analyses because they were strongly correlated (*r* ≥ 0.7) with other covariates. This reduced our suite of environmental covariates to 6: SNOW, TPI, BIOMASS, VEGCLASS, AGE, and CANCOV. The primary objective of our study was to compare the performance and reliability of SDMs generated for a rare, elusive, and cryptic forest carnivore using occurrence records that contrasted strongly in data quality. Accordingly, we used the default settings in Maxent for feature types (i.e., auto selection of linear, quadratic, product, threshold, and hinge features), regularization (a parameter that reduces over-fitting [31, 49]), and prevalence (proportion of the landscape occupied by the species [49]).

We used a multi-step process to generate and test Maxent models for each dataset. First, we randomly selected and set aside 20% of the verifiable fisher occurrence records (*n* = 25) to be used as an independent sample for testing the final models generated with each dataset. We then modeled each dataset (verifiable records *n* = 100; unscreened records *n* = 389) using bootstrapping with 100 replications in Maxent to generate jackknife tests for evaluating covariate contributions and training gain (a likelihood function). Starting with the full model for each dataset, we removed the weakest covariate and generated a new model. The weakest covariate was the one that decreased the training gain by the least amount compared to the full model when it was excluded from the model. We continued this process, each time removing the weakest covariate and generating a new model with the remaining covariates until only 1 covariate remained. The strongest model was the one that had the fewest covariates and did not exhibit a significant decrease in training gain (i.e., resulting 95% confidence intervals overlapped) compared to the model with the highest training gain (typically, the full model; [5, 50–51]). To evaluate model performance, we conducted a final Maxent analysis for each of the strongest models using the verifiable or unscreened fisher records as the training data, and our independent sample of 25 verifiable fisher records to test each model. We used several metrics to evaluate model performance including AUC_test_, training and test gains, and omission rate tests (1-sided tests of the null hypothesis that test localities are predicted no better than random). For presence-only data, AUC_test_ is the probability that a randomly selected presence location will be ranked higher than a randomly selected background location [6, 31, 49]. Models with AUC_test_ values > 0.75 are considered to contain useful information [4].

We used the logistic output in Maxent to produce distribution maps for the fisher in the Pacific States based on the strongest model from each data set. The logistic output is the relative probability of species occurrence conditioned on the environmental covariates [49, 52]; i.e., it represents a relative index of habitat quality [4, 31, 48]. To provide the best estimate of potential fisher distribution for visual interpretation, we created the map for the verifiable model using the full set of occurrence records (*n* = 125; see [6]). Our analysis area encompassed much of the Pacific States, yet our map pixel was relatively small (0.06 km^2^). To facilitate the interpretation of broad-scale patterns in the SDMs we generated, we pooled relative habitat quality indices into a small number of bins. Because our study animals and research objectives were similar and our analysis areas comparable in size, we followed Frey et al. [7] and binned habitat-quality indices into 4 habitat-quality classes: non-habitat (habitat-quality index = 0), low-quality habitat (indices > 0–0.2), medium-quality habitat (indices > 0.2–0.5), and high-quality habitat (indices > 0.5). We imported the logistic output from Maxent into ArcGIS to create the final species distribution maps, and to calculate mean values for the continuous covariates in each of the final models.

## Results

**Fig 2:**
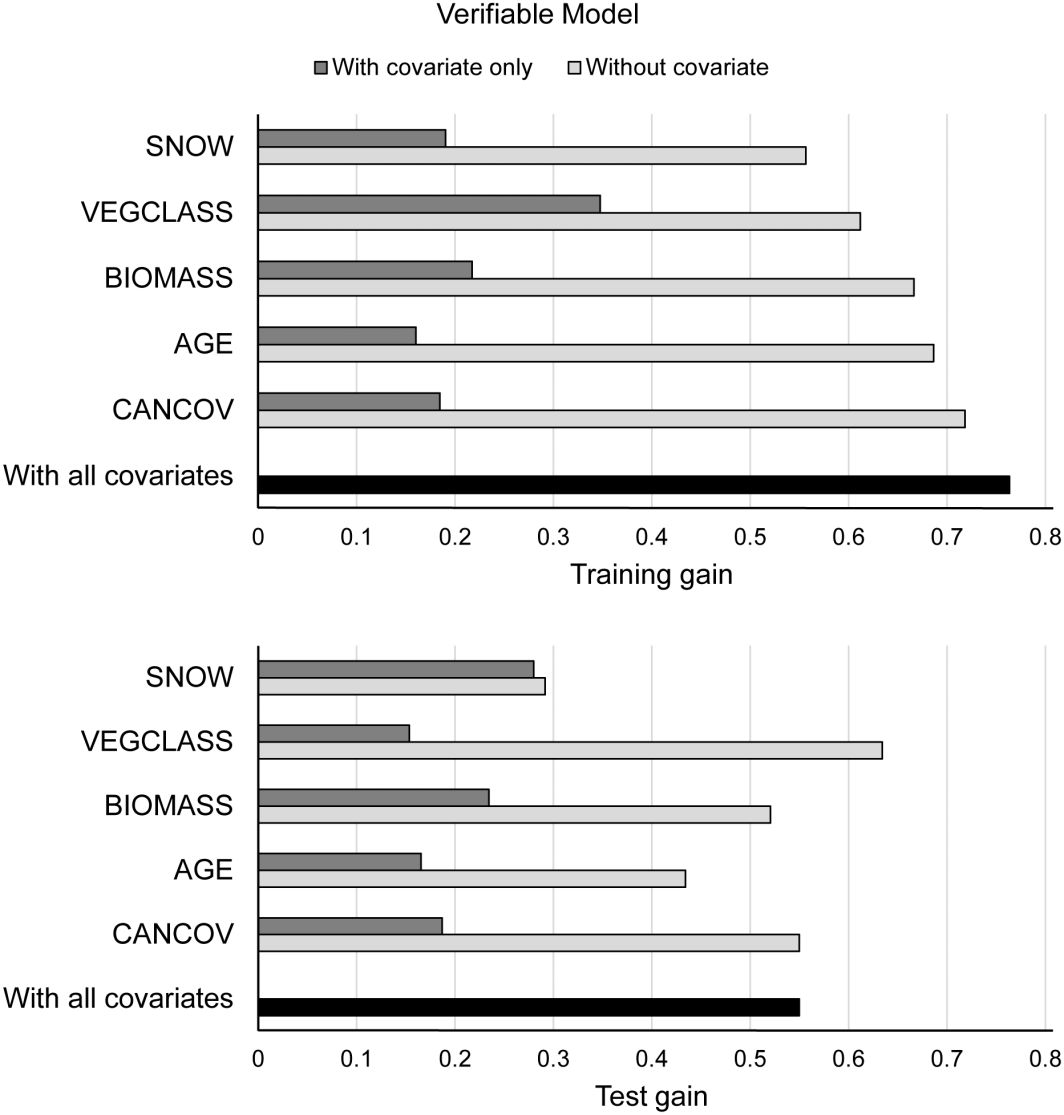
Results from jackknife tests of regularized training gain (upper graph) and test gain (lower graph) generated by running the final verifiable fisher distribution model in Maxent with an independent test sample.

Based on our bootstrapping process, the strongest and most parsimonious Maxent model generated with verifiable fisher occurrence records was a 5-variable model that included SNOW, VEGCLASS, BIOMASS, AGE, and CANCOV. The removal of TPI from the 6-variable (full) model did not significantly decrease the training gain, indicating that this covariate contributed little to model performance. The AUC_test_ value we generated by running the final 5-variable model with our independent test sample was 0.78, indicating that a random selection of presence locations would be ranked higher than a random selection of background locations 78% of the time. Furthermore, the mean likelihood of fisher occurrence (inverse log[training gain for the final model] or e^0.7635^) was 2.1 times greater than that of a random background location. Each of the 5 covariates in the final model contained unique information, especially SNOW, which resulted in the largest decrease in training gain when it was omitted from the model (Fig 2). When only 1 covariate was included, VEGCLASS fit the training data best (i.e., had the highest gain) followed by BIOMASS, SNOW, CANCOV, and AGE. In addition, our findings demonstrated that the verifiable model fit the independent test data well (Fig 2). The relative importance of SNOW in the test gain plot was greater than in the training gain plot, indicating that SNOW not only contributed the most unique information, but was also the most useful covariate for predicting the distribution of our test sample. Compared to the training data, VEGCLASS did not contribute as much to predicting the distribution of the test sample, whereas the relative importance of remaining covariates was similar between the training and test datasets. Lastly, binomial omission-rate tests were statistically significant (*P* ≤ 0.05) for 3 commonly used cumulative thresholds (1, 5, and 10).

**Fig 3:**
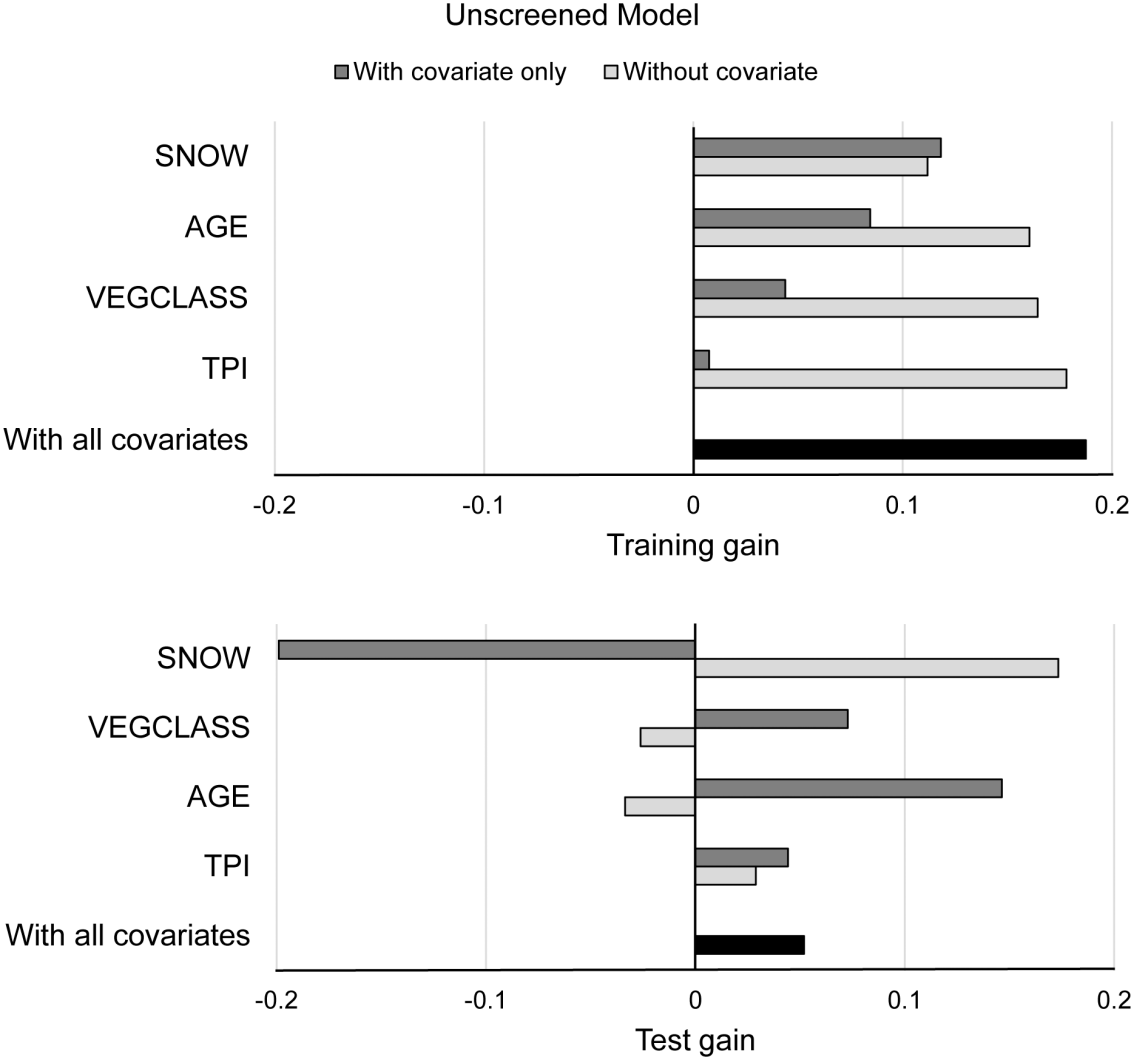
Results from jackknife tests of regularized training gain (upper graph) and test gain (lower graph) generated by running the final unscreened fisher model in Maxent with an independent test sample.

The strongest and most parsimonious model generated with unscreened fisher occurrence records was a 4-variable model that included SNOW, AGE, VEGCLASS, and TPI. The removal of BIOMASS and CANCOV from the full model did not significantly decrease the training gain, indicating that they contributed little to model performance. As in the final verifiable fisher model, SNOW was the most important covariate in the final unscreened model (Fig 3). However, the unscreened fisher model had an AUC_test_ value of 0.62, indicating that it did not contain useful information, and the likelihood of fisher occurrence based on the unscreened model was only 1.2 times greater than that of background locations, indicating further that the model was weak and a poor predictor of fisher occurrence. In addition, jackknife tests for test gain (Fig 3) demonstrated that the unscreened model performed poorly at predicting the distribution of our independent test sample. Although SNOW was the most important covariate for predicting the distribution of the unscreened records used for training, it did not contain any useful information for predicting the distribution of the test data (Fig 3). Lastly, in contrast to the verifiable fisher model, none of the omission tests for the unscreened model were statistically significant for cumulative thresholds of 1, 5, or 10.

**Fig 4:**
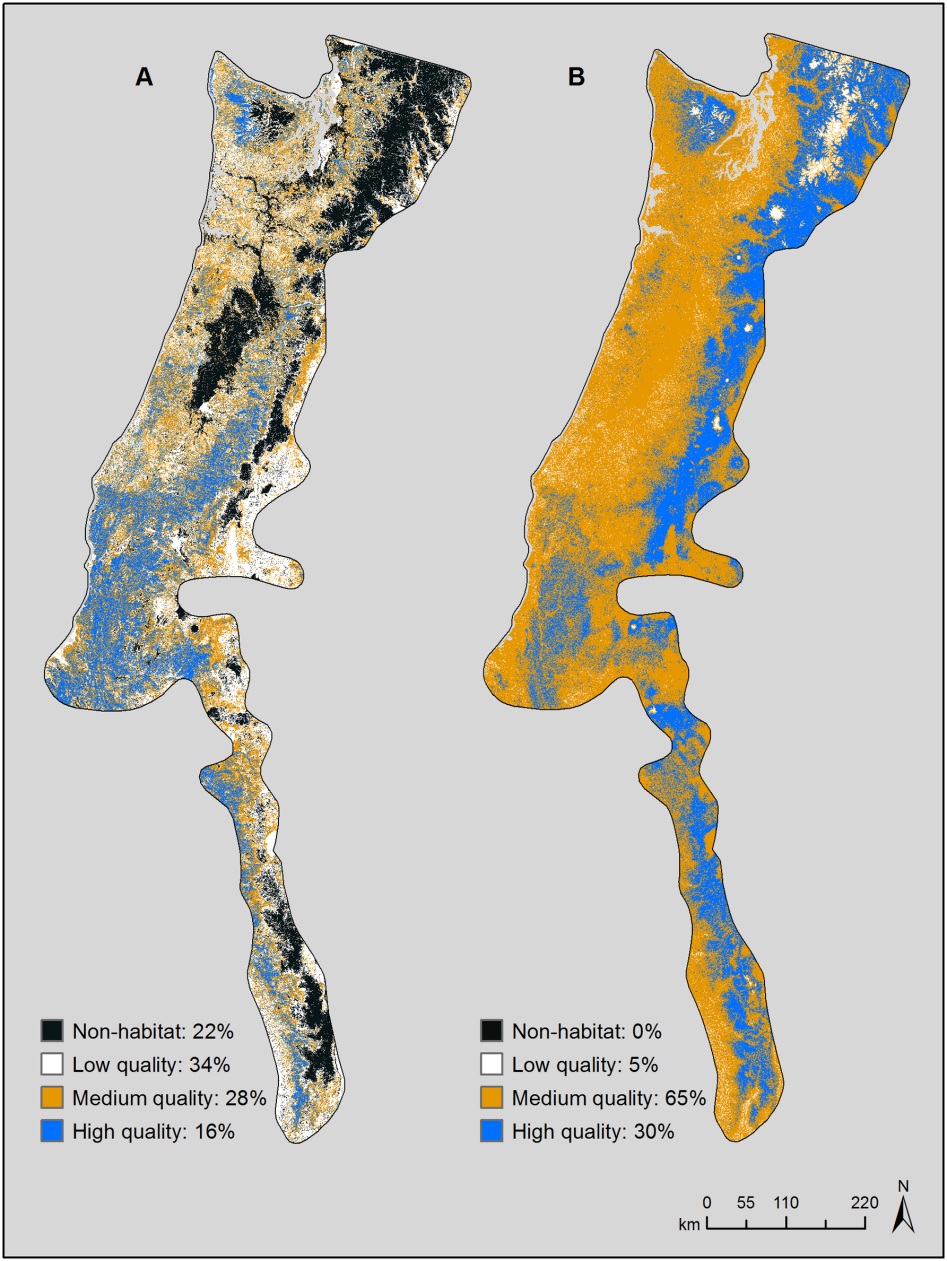
Species distribution maps for the fisher in the Pacific States created using logistic values (relative habitat-quality indices) generated in Maxent based on (A) the final verifiable fisher model, and (B) the final unscreened fisher model.

The fisher distribution map based on the final verifiable fisher model differed substantially from the distribution map based on the final unscreened fisher model (Fig 4). In the verifiable fisher map, high-quality habitat comprised 16% of the analysis area and was concentrated primarily in the western Olympic Mountains, the Cascade Range in southern Oregon, the Klamath Mountains in southern Oregon and northern California, and the western Sierra Nevada in California (Figs 1B and 4A). Medium- and low-quality habitats comprised 28 and 34% of the analysis area, respectively, and were scattered throughout the area. Non-habitat comprised 22% of the analysis area and was located primarily in interior valleys and at high elevations (> 1,524 m) in the Olympic Mountains, Cascade Range, and Sierra Nevada. In the unscreened distribution map, high-quality habitat comprised 30% of the analysis area and was located primarily at high elevations in the Olympics, Cascades, and Sierra Nevada (Figs 1B and 4B). Medium-quality habitat had the highest prevalence (65%), low-quality habitat comprised only 5% of the analysis area, and non-habitat was absent altogether.

**Fig 5:**
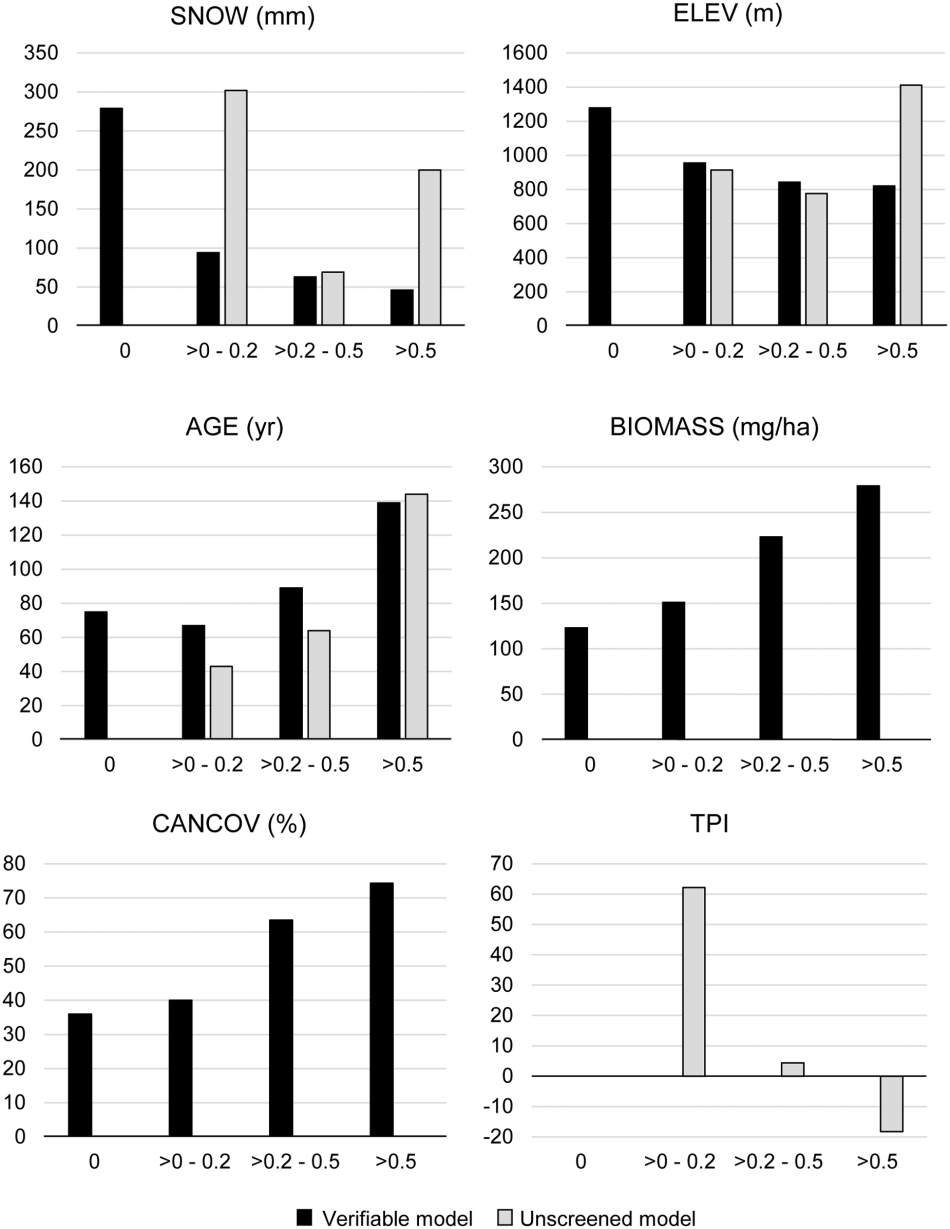
Mean values for continuous covariates included in the final verifiable and unscreened fisher distribution models by relative habitat quality classes. Elevation (ELEV) was not included as a covariate in the modeling process, but is presented here to help elucidate the contribution of SNOW to the final models.

Mean values for the continuous covariates that were included in both final distribution models (SNOW and AGE) differed in several ways. In the verifiable distribution map, habitat quality was inversely related to winter snowfall, and positively associated with stand age, biomass, and canopy cover (Fig 5). In the unscreened distribution map, winter snowfall was highest in low-quality habitat (Fig 5), but those habitat conditions represented only 5% of the analysis area (Fig 4B). For the remaining 95%, habitat quality was positively associated with winter snowfall. Thus, in the unscreened distribution map, habitat quality was positively associated with winter snow and stand age (Fig 5). The relation between habitat quality and topographic position was unclear, however, due to the poor performance of this model and the minor importance of this covariate (Figs 3 and 5). The only categorical variable used in our analyses (VEGCLASS) was included in both final models. For the verifiable model, habitat quality was positively associated with moderate to closed-canopy (≥ 40% canopy cover) conifer-dominated forests containing large trees (QMD > 50 cm) and with moderate to closed-canopy mixed forests (20–65% of basal area composed of hardwoods) with medium-sized trees (QMD = 25–50 cm). For the unscreened model, habitat quality was positively related to moderate to closed-canopy conifer-dominated forests with medium-sized trees.

## Discussion

Fishers and martens are often confused by observers [21, 25] because they are similar in shape, color, and size (especially female fishers and male martens), their ranges overlap in some areas, and both are secretive, semi-arboreal, and occur primarily in densely forested habitats with high-amounts of residual forest structures that provide denning, resting, and foraging habitat. In this region, however, fishers typically occur in low- to mid-elevation forests where deep, soft snow does not accumulate, because it is energetically demanding for them to travel in such conditions, and they cannot hunt effectively in the subnivean zone [21, 41–42]. Although remnant marten populations occur in coastal areas of the Pacific States that receive little snowfall [24], most marten populations are restricted to high-elevation montane forests where deep snowpacks form. Unlike fishers, martens hunt winter-active small mammals effectively within the subnivean zone, and their movements are not restricted by snow conditions [24, 30, 53]. Although fishers and martens generally have contrasting relations with elevation and winter snowfall, both species are associated primarily with closed-canopy, conifer-dominated, mesic forests that contain abundant snags, decadent trees, and logs [54]. Such conditions are most likely to occur in older forests, as our results indicated for both verifiable and unscreened models (Fig 5).

The fisher distribution map based on the final verifiable model differed in many substantive ways from the map based on the final unscreened model (Fig 4). Although it was based on occurrence records that were limited in geographic extent (Fig 1A), the verifiable fisher model was statistically strong and produced a compelling species distribution map for the fisher in the Pacific States (Fig 4A). Relatively large and contiguous areas of high-quality fisher habitat occur at relatively low elevations (< 915 m) in the western Olympic Mountains in Washington, the southern Coast Range and western portions of the southern Cascade Range in Oregon, the Klamath Mountains in Oregon and California, and western portions of the Sierra Nevada in California (Figs 1B and 4A). These areas correspond closely to the current distribution of fishers in the Pacific States [23]. In addition, the verifiable fisher model is consistent with previous habitat modeling by the state of Washington, which indicated that the Olympic Mountains contained the largest contiguous blocks of high-quality fisher habitat in western Washington [29].

In the verifiable fisher map, non- and low-quality habitats occur primarily in areas that fishers did not occupy historically, such as high-elevation (> 1,524 m) montane regions, interior valleys and oak savannas, and areas of human settlement (Figs 1B and 4A) [21, 37]. In other portions of the analysis area in Washington and Oregon, including many lower elevation forests that supported fishers historically but no longer do [21], the model contains few large, contiguous areas of high-quality fisher habitat; rather, low- and medium-quality fisher habitats are common, widespread, and interspersed with relatively small patches of high-quality fisher habitat. Lastly, as would be expected for a habitat specialist [21, 37], high-quality fisher habitat is restricted in distribution (16%), and medium- and low-quality fisher habitat increases in prevalence (28 and 34%, respectively) as habitat quality decreases (Fig 4A).

In contrast, the final unscreened fisher model was statistically weak and the resulting distribution map (Fig 4B) strongly contradicted our understanding of the distribution of fishers in the Pacific States. In particular, the unscreened distribution map depicts many large, contiguous areas of high-quality habitat at high elevations (> 1,524 m) in the Olympic Mountains, Cascade Range, and Sierra Nevada (Figs 1B and 4B), including subalpine forests, open parklands, and alpine meadows at or above tree line, none of which supported fisher populations historically [21, 37]. Non-habitat is absent, and low-quality habitat is relatively rare (5%) and restricted primarily to areas of permanent ice and snow (Fig 4B).

The most influential covariate in both final models was winter snowfall, yet patterns in each distribution map differed strongly (Fig 5). Although it was not used in the modeling process, we included mean values for elevation (ELEV) in Fig 5 to help elucidate results for winter snowfall. In the verifiable distribution map, habitat quality was inversely related to both snowfall and elevation, but positively associated with stand age, biomass, and canopy cover (Fig 5). The most influential vegetation classes were those dominated by large conifers or mixed conifer/hardwood forests with medium-sized trees. Altogether, these patterns conform closely to our understandings of the fisher’s environmental relations in this region [21, 37, 41–42, 53]. In contrast, the best habitat in the unscreened distribution map was located in relatively old (> 140 yr), conifer-dominated forests at high elevations (mean = 1,412 m) where deep snowpacks form (Fig 5).

In addition to being more spatially extensive, the unscreened occurrence records have a broader temporal range than the verifiable records (1954–1993 and 1989–2001, respectively; Table 1), which could have confounded our interpretations. During the second half of the 20^th^ century, most low-elevation forests with high productivity in this region were modified by human activities or natural disturbances. However, the association between habitat quality and stand age was similar in both distribution maps. Thus, alterations in low-elevation forests from logging, agricultural conversion, urbanization, or natural disturbances do not appear to be an important source of between-model differences. Based on our knowledge of the fisher’s ecological relations, abiotic associations, and current distribution in this region, the verifiable distribution map represents an accurate and reliable assessment of the status of fisher habitat at the regional scale in the Pacific States. The unscreened fisher distribution map does not fit these patterns; rather, it primarily reflects our expectations regarding the distribution of Pacific martens in the analysis area.

Accordingly, we believe the differences in these distribution maps result primarily from a high proportion of misidentifications of martens in the unscreened dataset. Standardized remote-camera surveys targeting fishers were conducted throughout their historical range in the Pacific States during the 1990s (see Fig 2b in [16]). These surveys only detected fishers in southwestern Oregon, and northwestern and southern California; i.e., the areas represented by verifiable occurrence records in Fig 1A. Misidentifications are common in anecdotal occurrence records, because they are often based on fleeting glimpses made by individuals who are unfamiliar with the animal they claim to have seen; in such cases, species identifications are usually determined using a field guide and the process of elimination ([15]; K. Aubry, unpublished data). Because the unscreened model was statistically weak, devoid of non-habitat, and contained substantially larger areas of medium- and high-quality habitat than the verifiable model, it is likely that the unscreened dataset was also contaminated with misidentifications of other forest carnivores, especially the American mink (*Mustela vison*) and northern river otter (*Lontra canadensis*) [21], which occupy a much broader range of elevations and forest conditions than the fisher or Pacific marten.

The occurrence records we used to build our verifiable fisher model contained few (if any) misidentifications, but were limited in geographic extent (Fig 1A). Nonetheless, those data generated a strong species distribution model for the fisher throughout the Pacific States that aligned closely with our understandings of their current distribution and environmental relations in this region. Accordingly, our findings provide additional evidence that fisher habitat relations are driven largely by forest structural conditions and are strikingly consistent throughout their range in the Pacific States, as Aubry et al. [38] reported. Consequently, accurate species identifications are far more important than representative sampling for generating reliable distribution models for the fisher in the Pacific States. Although the verifiable fisher model we generated is both statistically and biologically strong, it was developed to depict broad-scale patterns in fisher habitat quality throughout the Pacific States. Accordingly, it is not appropriate to use our verifiable fisher map to evaluate patterns in potential fisher habitat at finer spatial scales.

Our findings demonstrate that for rare, elusive, and cryptic species that are easily confused with more common and broadly distributed species, it is essential practitioners ensure that misidentifications are rare or non-existent when generating species distribution models from occurrence records. Such misidentifications are particularly insidious in a conservation context, because as the target species declines in abundance relative to the contaminating species, the proportion of false positives will increase [17]. We strongly believe that anecdotal occurrence records should not be used to generate species distribution models, but if such data are used in modeling efforts, resulting models should be tested with verifiable occurrence records to ensure that the dataset has not been contaminated with misidentifications of other species.

## Supporting information

S1 Appendix Geographic coordinates (Albers, NAD 1983) of verifiable and unscreened fisher occurrence records from the Pacific coastal states (1954–2001) used in our analyses. (XLSX)
